# Miscellaneous

**Published:** 1888-10

**Authors:** 


### MISCELLANEOUS.

Intelligence of a Horse.—A novel story comes from Vandeburg county. A day or two ago a horse was standing tied to a fence in the yards of the Sunny Side Coal Company, in that county. A drunken man was staggering around that neighborhood, and in a moment of drunken frenzy took out his pocket knife, and seeing no one around on whom to vent his spleen, he walked up to the horse and deliberately plunged the blade into the dumb brute's neck. The gash was a long one and quite severe, and the blood flowed from the wound profusely. The horse writhed in agony, and in its struggles broke the hitching rein and ran out of the lot.

The horse kept up his speed down the road until it came to a drug store on Fulton street, owned by Jenkins & Kyle. The animal stalked into the store deliberately, and went as far back as the prescription case, and set up a most pitiful neighing. The clerk was alarmed, but spoke gently to the animal, and taking a sponge, bathed the ugly wound in cold water, much to the relief of the brute. The proprietor, Mr. Kyle, then sewed up the wound and tied a band around the animal's neck. The horse was then led back to the yards, seemingly happy and contented. Mr. Kyle is positive in the assertion that this is the neatest case of brute sagacity on record, and points to the blood spots on the floor of his store as proof.

Our Seventy-Year Clocks.—Our brains are seventy-year clocks. The Angel of Life winds them up once for all, then closes the case and gives the key into the hand of the Angel of Resurrection.

Tic-tac ! tic-tac ! go the wheels of thought. Our will cannot stop them. They cannot stop themselves. Sleep cannot still them. Madness only makes them go faster. Death alone can break into the case, and, seizing the ever-swinging pendulum, which we call the heart, silences at last the clicking of the terrible escapement we have carried so long beneath our wrinkled foreheads.

If we could only get at them, as we lie on our pillows and count the dead beats of thought after thought and image after image jarring through the over-tired organ ! Will nobody back those wheels, uncouple the pinion, cut the string that holds those weights, blow up the infernal machine with gunpowder ? What a passion comes over us sometimes for silence and rest ! that this terrible mechanism, unwinding the endless tapestry of time, embroidered with spectral figures of life and death, could have but one brief holiday ! Who can wonder that men swing themselves off from beams in hempen lassos ? that they jump off from parapets into the swift and gurgling waters beneath ? that they take counsel of the grim friend who has to utter but his one peremptory monosyllable, and the restless machine is shivered as a vase that is dashed upon a marble floor ? * * * * If anybody would only contrive some kind of a lever that one could thrust in among the works of this horrid automaton and check them or alter their rate of going, what would the world give for the discovery ?—O. W. Holmes.

Principles of Cream Raising.—Milk does not become unmanageable if the temperature of the room does not rise above sixty-five degrees in the middle of the day. It should be borne in mind always that this question of temperature, closely followed by those of cleanliness, watchfulness and industry, is of very great importance in the dairy. No dairy equipment is complete without a thermometer. The colder the room, especially in the summer, the faster and more thoroughly the cream will rise. This is the result of natural laws. Water, of which milk is chiefly composed, shrinks sooner than fat does under the influence of heat. This is because it is a better conductor of cold and heat than fat is. The result of milk being placed in an atmosphere or in water much colder than the milk, is seen in the comparatively rapid ascent of the cream. This is simply because cream—the fatty globules—being a slower conductor of cold than water is, retains its buoyancy longer, and so rises to the surface quicker in a falling temperature of the milk then it does in a stationary one. This is one of the prime secrets of the success of the various cream raising cans which are either submerged or set deep in cold water, and that style of cans which enables the entire body of milk to be cooled the most quickly will naturally raise the most cream in the least time.

THE STEVENS' IMPEROYAL FLOUR has become the most popular in the market. Genuine merit—and avertising in HALL'S JOURNAL OF HEALTH.

### WHO CAN TELL?

BY HORACE M. RICHARDS.

Who can tell save God the giver
What the life "Beyond the river!"
When I lay its burthens down,
Bear I cross, or wear I crown?

What the fruit of toil and pain?
Is it loss? or is it gain?
What the harvest "over there?"
Is it joy: or dark despair?

Here below I burthens bear—
Will they lighten "over there?"
Here below I feel oppressed—
"Over there," will I find rest?"

While we walk in earthly garb,
Hearts are pierced with sorrows barb,
When we don the spirits dress—
Feel we still earth's sore distress?

Here below is gloom and night,
Find we there a radiance bright?
Who can tell save God the giver,
What the life, "beyond the river?"

